# Cerebral Abscess Associated With Odontogenic Bacteremias, Hypoxemia, and Iron Loading in Immunocompetent Patients With Right-to-Left Shunting Through Pulmonary Arteriovenous Malformations

**DOI:** 10.1093/cid/cix373

**Published:** 2017-04-19

**Authors:** Emily J. Boother, Sheila Brownlow, Hannah C. Tighe, Kathleen B. Bamford, James E. Jackson, Claire L. Shovlin

**Affiliations:** 1 Cardiovascular Translational Sciences, National Heart and Lung Institute and; 2 Imperial College School of Medicine, Imperial College London, and; 3 Respiratory Medicine,; 4 Microbiology, and; 5 Department of Imaging, Imperial College Healthcare NHS Trust, London, United Kingdom

**Keywords:** hereditary hemorrhagic telangiectasia, intravenous iron, transferrin saturation index, oxygen, hypoxemia

## Abstract

**Background:**

Cerebral abscess is a recognized complication of pulmonary arteriovenous malformations (PAVMs) that allow systemic venous blood to bypass the pulmonary capillary bed through anatomic right-to-left shunts. Broader implications and mechanisms remain poorly explored.

**Methods:**

Between June 2005 and December 2016, at a single institution, 445 consecutive adult patients with computed tomography–confirmed PAVMs (including 403 [90.5%] with hereditary hemorrhagic telangiectasia) were recruited to a prospective series. Multivariate logistic regression was performed and detailed periabscess histories were evaluated to identify potential associations with cerebral abscess. Rates were compared to an earlier nonoverlapping series.

**Results:**

Thirty-seven of the 445 (8.3%) patients experienced a cerebral abscess at a median age of 50 years (range, 19–76 years). The rate adjusted for ascertainment bias was 27 of 435 (6.2%). Twenty-nine of 37 (78.4%) patients with abscess had no PAVM diagnosis prior to their abscess, a rate unchanged from earlier UK series. Twenty-one of 37 (56.7%) suffered residual neurological deficits (most commonly memory/cognition impairment), hemiparesis, and visual defects. Isolation of periodontal microbes, and precipitating dental and other interventional events, emphasized potential sources of endovascular inoculations. In multivariate logistic regression, cerebral abscess was associated with low oxygen saturation (indicating greater right-to-left shunting); higher transferrin iron saturation index; intravenous iron use for anemia (adjusted odds ratio, 5.4 [95% confidence interval, 1.4–21.1]); male sex; and venous thromboemboli. There were no relationships with anatomic attributes of PAVMs, or red cell indices often increased due to secondary polycythemia.

**Conclusions:**

Greater appreciation of the risk of cerebral abscess in undiagnosed PAVMs is required. Lower oxygen saturation and intravenous iron may be modifiable risk factors.

## Summary

In a UK series, 37 of 445 (8.3%) patients with pulmonary arteriovenous malformations (PAVMs) experienced a cerebral abscess, often pre-PAVM diagnosis as first presentation. Odontogenic organisms, dental interventions, lower oxygen saturation, iron loading, and intravenous iron use were associated with abscess risk.

Cerebral abscess is a recognized risk for immunocompetent patients with cyanotic congenital heart disease and intracardiac right-to-left shunts [1]. Pulmonary arteriovenous malformations (PAVMs) also provide a right-to-left shunt, through abnormal vascular communications between pulmonary arteries and pulmonary veins [2, 3]. PAVMs are estimated to affect as many as 1 in 2600 people [4], but are subject to substantial underascertainment [5]. Although recognized to cause cerebral abscess for >50 years [6], the causal link seems poorly appreciated [7], compounded by low diagnostic rates of PAVMs at the time of cerebral abscess. In our previous series, the majority of cerebral abscesses occurred prior to PAVM diagnosis, with a median 2-year delay between the abscess and later PAVM diagnosis [8].

PAVMs impair gas exchange: Hypoxemia is common and directly proportional to the fraction of pulmonary arterial blood transiting the PAVMs [2, 9]. Hematological and hemodynamic compensatory responses enable patients to compensate for lower blood oxygenation [10–12], and affected individuals often remain undiagnosed for decades. Irrespective of respiratory symptoms [8], patients with PAVMs remain at major risk of paradoxical emboli: Ischemic strokes clinically affect >10% of all series, with a greater subclinical burden of arterial occlusion [9, 13]. Recent series suggest that cerebral abscesses affect between 7.8% and 9% of PAVM patients [2]: These events are attributed to impaired pulmonary filtration of thromboembolic material that exceeds the normal pulmonary capillary diameter of 7–10 μm [14]. PAVM treatment is recommended to reduce these neurological risks, and this is usually performed by embolization therapy [3, 15]. Unfortunately, PAVMs are often technically too small and/or numerous for embolization, and many treated patients are left with residual right-to-left shunts [2, 3].

PAVMs can occur sporadically, but also affect at least 50% of people with hereditary hemorrhagic telangiectasia (HHT) [16–18]. HHT is inherited as an autosomal dominant trait, most commonly resulting from a pathogenic sequence variant in *ENG*, *ACVRL1*, or *SMAD4* [19]. HHT-associated PAVMs represent the majority of PAVMs reported in medical series, in part due to PAVM screening programs in HHT populations. PAVM patients with HHT have concurrent medical issues that might be predicted to have an impact on cerebral abscess pathogenesis. Excepting PAVM-associated abscesses, there is no overt clinical association with immunodeficiency or conventional inflammation. Instead, HHT is characterized by recurrent bleeding from abnormal vascular structures, and iron deficiency anemia due to underreplacement of hemorrhagic iron losses [20]. At least 1 in 3 HHT patients require long-term oral iron supplements, with smaller proportions requiring intravenous iron and/or blood transfusions.

For PAVM patients, microbial isolates [8, 21] and documented preceding dental interventions [8, 21; and case reports] have implicated periodontal organisms in cerebral abscess pathogenesis. Patients are advised to maintain good dental hygiene, and to use oral prophylactic antibiotics before dental and surgical procedures [22]. With recent evidence implying that intravenous administration of antibiotics is more effective in preventing dental bacteremias [23], identifying which PAVM patients are particularly prone to cerebral abscess becomes more important. Neurological complications are less frequent with low-grade, potentially functional intrapulmonary right-to-left shunting [24], as commonly found in HHT patients with no evidence of PAVMs on computed tomographic scans [25] and in the general population [26]. But for patients with radiographic evidence of PAVMs, other than those with prior occurrence of cerebral abscess, to date it has been very difficult to identify which patients are at greater risk.

Our aims were to evaluate whether previously recommended measures (embolization of asymptomatic PAVMs, judicious dental hygiene, and antibiotic prophylaxis prior to dental and surgical procedures [8]) had resulted in reduced morbidity from cerebral abscess, and to identify particularly high-risk patients suitable for greater targeting of preventive measures.

## MATERIALS AND METHODS

### Subject Evaluations

The study was ethically approved by the Hammersmith, Queen Charlotte’s, Chelsea, and Acton Hospital Research Ethics Committee (LREC 2000/5764). Patients with radiologically diagnosed PAVMs presenting for the first time between June 2005 and December 2016 were recruited prospectively and consecutively at the time of presentation. All reported evaluations were performed as part of routine clinical care, as detailed in the Supplementary Data.

In brief, symptoms/complications from PAVMs (including prior cerebral abscess), symptoms/complications of HHT, and full clinical histories were recorded. Clinical examination included evaluation of HHT and dental hygiene. The mean oxygen saturation (SaO_2_) after standing for 7–10 minutes was recorded, as this better reflects right-to-left shunt size than other postures [9, 10]. Patients received written advice on optimizing dental hygiene and prophylactic antibiotic use prior to dental and surgical procedures. Where indicated, PAVM embolization was performed [3] and the diameter of feeding arteries to the PAVM sac(s), and pulmonary artery pressures measured.

A diagnosis of HHT was made in the presence of PAVMs plus at least 2 of epistaxis, characteristic telangiectasia, family history, and other visceral AVMs, or a positive HHT gene test [16–19]. All patients were offered screening/investigation for iron deficiency anemia (using complete blood counts, serum iron, transferrin saturation index [T*f*SI], and ferritin). Patients with symptoms suggestive of other visceral AVMs underwent relevant investigations, but formal screening was restricted to selected cases.

### Data Analyses

Patients were initially assigned to abscess/nonabscess groups based on presentation data. If a subsequent cerebral abscess was reported, prior to data analysis, patents were reassigned to the abscess group. Case notes of those who had experienced a cerebral abscess were retrospectively evaluated to additionally capture patient-specific variables at the closest timepoint to the abscess (the interval ranged from 9 months preabscess to 480 months postabscess; median, 9 months postabscess). “Nonabscess” patients theoretically could go on to develop an abscess at any point up to their death, so their data were censored at presentation. Presentation data therefore usually reflected the “pretreatment” period of PAVM history, but as detailed in the Supplementary Data, 31 patients had received PAVM treatment prior to assessment/blood tests. Where only posttreatment data were available, SaO_2_, hemoglobin, and red cell indices were recorded as unknown, to minimize bias by assigning spuriously normal indices to a patient who may have previously had decades of life with untreated PAVMs and different risk factor status.

Oxygen content of arterial blood (CaO_2_) was calculated as 1.34 × hemoglobin × SaO_2_/100 [27]. Logistic regression analyses used cerebral abscess as the outcome (dependent) variable for 34 variables: *P* values were also calculated by postestimation Wald tests, and final models confirmed using receiver operating characteristic analyses.

## RESULTS

### Cerebral Abscess Rate

The 445 consecutive adult PAVM patients were aged 16–89 years (median, 48 years). Two hundred sixty-seven (60%) were female. Thirty-seven (8.3%) experienced a cerebral abscess with the diagnosis confirmed by neurosurgical drainage: 32 prior to study inclusion, and 5 after study inclusion and provision of antibiotic advice (4 of these had also received embolization). In 10 (27%) cases, PAVMs were diagnosed because of the cerebral abscess. Excluding these individuals, to correct for ascertainment bias, the adjusted rate was 6.1% (95% confidence interval [CI], 3.9%–8.5%).

Diagnostic demographics were compared to the series of previously reported, nonoverlapping patients [8] (Table 1). The crude abscess rate appeared to be lower (*P* = .07), but the 2 series became more comparable when adjusted for ascertainment bias. Age at abscess, patient sex, and proportions with a prior PAVM diagnosis or postabscess residual neurological deficit did not differ between the series. The current cohort exhibited a shorter interval between experiencing a cerebral abscess and receiving a PAVM diagnosis (Table 1).

**Table 1.** Comparative Demographics Between Current and Previous Pulmonary Arteriovenous Malformation/Cerebral Abscess Series

Characteristic	Current Series	Previous Series^a^	Combined	*P* Value^b^
PAVM cases, No.	445	219	664^c^	…
Date of first institutional review	June 2005–Dec 2016	April 1984–May 2005	…	…
All cerebral abscess cases^d^	37 (8.3)	28 (12.8)	65 (9.8)	.07
Male sex	14 (38)	17 (60.7)	31 (47.7)	.08
Abscess rate adjusting for ascertainment bias	27/435 (6.2)	19/210 (9.05)	46 (7.1)	.26
Age at abscess, median (*Q*1, *Q*3), y	48 (34, 62)	44 (32.5, 50.5)	44 (32, 52)	.89
PAVM diagnosis prior to abscess^e^	8 (21.6)^f^	10 (35.7)	18 (27.7)	.27
PAVM diagnosis following abscess	29 (78.4)	18 (64.3)	47 (72)	.27
Abscess-to-PAVM diagnosis interval, mo, median (*Q*1, *Q*3)	0.5 (0.4, 1.0)	24 (0.0, 96.0)	0.5 (0.4, 27)	.01
No HHT diagnosis prior to abscess	27 (79.4)	17 (60.7)	44 (70.9)	.18
Permanent neurological deficit	19 (51.4)	17 (70.8)	36 (55.4)	.62

Data are presented as No. (%) unless otherwise indicated.

Abbreviations: HHT, hereditary hemorrhagic telangiectasia; PAVM, pulmonary arteriovenous malformation.

^a^Previously reported series [8].

^b^Categorical *P* values calculated by Fisher exact test, continuous values by Mann-Whitney test.

^c^No individual could be in both series.

^d^Cerebral abscess could occur at any time point until the end of the respective individual series (December 2016; May 2005) but, in the majority of cases, occurred prior to the first presentation to our institution.

^e^Seven had been previously treated a median of 5 years (range, 0–18 years) earlier, and 1 patient had declined treatment. Three patients experienced further abscesses (cerebral [n = 2], lung/chest [n = 2], and spinal [n = 1]).

^f^Eight (21.6%) described respiratory symptoms (most commonly dyspnea), but these were only sufficient to precipitate PAVM diagnosis in 3 cases.

### Morbidity Associated With Cerebral Abscess

**Figure 1.**
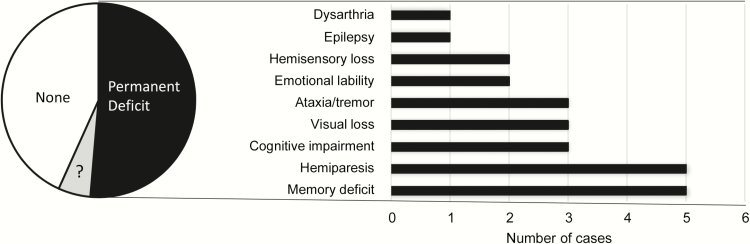
Persistent neurological deficits in the 37 cerebral abscess cases. Indirect neurological consequences (eg, postabscess strokes) are not included, but would increase the number of visual loss cases by 1. There were no fatalities in this group, but 3 patients with pulmonary arteriovenous malformation due to hereditary hemorrhagic telangiectasia had first-degree relatives who died as a direct result of a cerebral abscess. “?” represents cases where the final neurological outcome was not yet known.

All 37 patients required neurosurgery, approximately 6 weeks of intravenous antibiotics, prolonged inpatient hospital stays, and, in many cases, further rehabilitation following hospital discharge. Residual life-changing neurological deficits were recorded for 19 of 37 (51.4%) patients, most commonly memory loss or other cognitive impairment (8/37 [21.6%]); hemiparesis (5/37 [13.5%]); and/or visual field loss/blindness (3/37 [8.1%]) (Figure 1).

### Bacterial Isolates

As in previous series [8, 21], the majority of abscess cultures were culture-negative. The principal isolates cultured from the cerebral abscesses were microaerophilic and anaerobic bacteria predominantly of periodontal origin (Table 2).

**Table 2.** Bacterial Species Cultured From the Pulmonary Arteriovenous Malformation Patients With Cerebral Abscesses

Species	Current Cohort		Previous Cohort [9]^a^		Total Isolates	
	Cases	Associations^b^	Cases	Associations	Cases	% of 24 Positive Isolates
Streptococcal spp	4	3/4 dental	5	4/5 dental	9	41
*Streptococcus milleri*	1	Scale and polish, poor dental hygiene^c^	3	Post scale and polish (2 cases)	4	18^d^
*Streptococcus anginosus*	1	Ongoing major dental work	1	Very poor dental hygiene	2	9
Nonhemolytic streptococci	1	Deep gum pocket periodontitis	…	…	1	5
α-Hemolytic streptococci	1	None recorded	…	…	1	5
Uncharacterized	…	…	1	Very poor dental hygiene	1	5
*Actinomyces* spp^e^	2	Extraction, poor dental hygiene	2	Dental plates^f^	4	18
*Staphylococcus intermedius*	1	Recent dental work	…	…	1	5
Unspecified anaerobe species	1	Scale and polish, poor dental hygiene^c^	…	…	1	5
Bacteroides spp	…	…	2	Dental plates^f^	2	9
*Propionobacterium*	…	…	1	Dental plates^f^	1	5
*Porphyromonas*, *Gemella*, and *Peptostreptococcus*	…	…	1	Dental work and fillings	1	5
MRSA, *Enterococcus*	…	…	1	Recent venous access	1	5
Uncharacterized gram-positive rods	…	…	2	Poor dentition, dental abscess	2	9
Uncharacterized gram-positive cocci	…	…	1	Dental abscess	1	5

Abbreviation: MRSA, methicillin-resistant *Staphylococcus aureus*.

^a^Microbiological isolates include those from 4 additional abscesses in the previous cohort [8] that occurred after study closure, and thus were not included in either abscess series.

^b^All evident at the time or recalled within 12 months of intervention.

^c^Same case.

^d^Note that in 118 pediatric cases presenting to 4 UK neurosurgical centers over 12 years, this was the most frequent organism (38% of positive cultures), except after penetrating head injury or neurosurgery, for which *Staphylococcus aureus* was most common [30].

^e^ *Actinomyces israelii*, *Actinomyces meyeri*, and unspecified.

^f^Same case.

### PAVM Characteristics

PAVM characteristics varied in terms of anatomy (single or multiple); size of the largest feeding artery diameter; severity of right-to-left shunting (reflected by SaO_2_), and technical suitability for embolization therapy. At the time of the cerebral abscess, 36 (97.3%) patients had at least 1 PAVM that was technically large enough to be treated, although 5 (13.5%) had all PAVM feeding arteries with a diameter ≤3 mm—that is, at or below the limits of what is commonly considered treatable. At least 26 (70.2%) had additional PAVMs that were technically too small for embolization. Unfortunately, 31 (83.7%) had their abscesses prior to diagnosis of their PAVM(s).

### PAVM Patient Characteristics

Patient characteristics reflected the presence of PAVMs, commonly associated HHT, and other PAVM/HHT-independent pathologies. Only 1 individual displayed significant leukopenia (Supplementary Table 1). Three-quarters of the cohort were significantly hypoxemic at presentation, but maintained CaO_2_ by secondary erythrocytotic/polycythemic responses (Supplementary Table 1). At least 34 (91.8%) of the population had HHT, and due to chronic HHT blood losses, high proportions were iron deficient, used oral or intravenous iron regularly, and/or required blood transfusions. Hemoglobin values ranged from anemic to polycythemic values, reflecting the relative (and sometimes simultaneous [10, 11, 20]) influences of iron deficiency and secondary erythrocytosis.

### Periabscess Histories

Fourteen of the 37 patients with cerebral abscess (37.0%) described dental healthcare access, or untreated dental infections, in the months prior to abscess (Table 3). There was 1 case where direct extension was considered a potential pathogenic route. Less frequently, nondental potential sources of bacteremias were recorded in the weeks prior to the abscesses (Table 3). Unexpectedly, for 4 of 37 (10.8%) patients, the abscesses occurred while on short holidays abroad, and 5 (13.5%) patients reported very severe migraines in the days before the abscess.

**Table 3.** Timing of Interventional and Dental Histories for the 37 Patients With Cerebral Abscess

Event/State	No. of Cases (2005–2016 Series)	Range of Timepoints (Months Previously)
Dental infections	3	
Dental abscess(es)	3	Ongoing
Deep gum pocket periodontitis	1	Ongoing
Dental intervention^a^	11	
Scale and polish	4	0.5–3 (mean, 1.6)
Dental extraction	2	6–12 (mean, 9)
Root canal treatment	1	5
Occlusive braces	1	Ongoing ≥12 mo, poor hygiene
Other dental work	3	Ongoing, 0.25 mo and “recent”
Bronchoscopy	1	1
Obstetric surgery^b^ and epidural	1	1
Intravenous access	3	≥1 wk

^a^Seven individuals in dental intervention groups were also recorded on clinical examination at the time, or within 1 year, to have poor or very poor dental hygiene. Only 1 of the patients with a known diagnosis of pulmonary arteriovenous malformation used prophylactic antibiotics as recommended [8, 22].

^b^Cesarean delivery.

### Comparisons With Full PAVM Cohort

**Figure 2.**
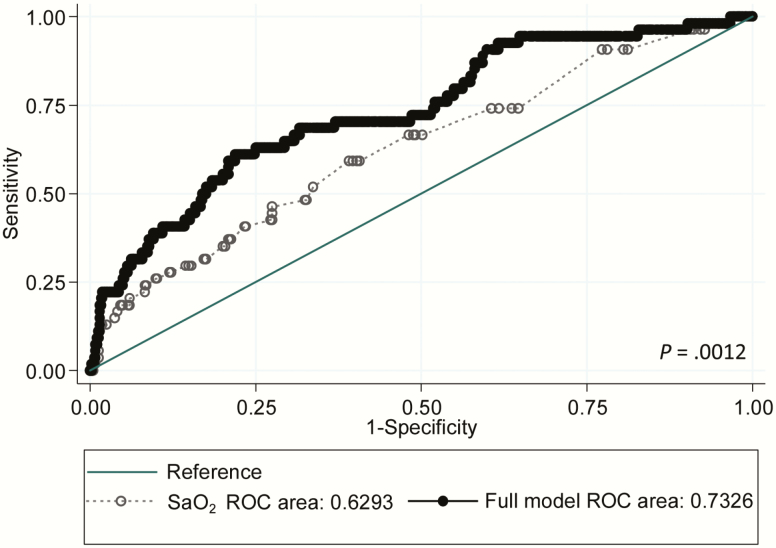
Comparison of the cerebral abscess risk receiver operating characteristic (ROC) model from oxygen saturation (SaO_2_) alone (dotted line/open symbols) and final model with SaO_2_, sex, transferrin saturation index, intravenous iron, and venous thromboembolus (solid black line/symbols). The 2 models provide areas under the curve of 0.63 and 0.73, respectively (*P* = .0012). Inclusion of feeding artery diameter marginally reduced the strength of the models, and the association was negative, implying that cerebral abscess were marginally more common for pulmonary arteriovenous malformations with smaller feeding artery diameters, once adjusted for other components of the model.

Univariate analyses suggested that patients with cerebral abscess were more likely to have lower SaO_2_, multiple PAVMs, higher platelet volumes, use intravenous iron, and/or suffer a venous thromboembolus (VTE) than nonabscess patients (Table 4). Surprisingly, there was no relationship between cerebral abscess risk and other PAVM neurological complications (ischemic stroke, migraine headaches), variables associated with ischemic stroke risk (low serum iron, high fibrinogen, or low pulmonary artery pressure [8, 9]), or largest PAVM feeding artery diameter (Table 4). In multiple regression analyses, adjusting for SaO_2_, the association of cerebral abscess with VTE persisted, but the associations with multiple PAVMs and platelet volume became less significant, and further relationships, particularly with T*f*SI, became evident. Table 5 and Figure 2 present the final model that explained the greatest proportion of biological variance.

**Table 4.** Evaluations at Assessment Closest to Abscess

Variable	No.	(%)		No.	(%)		*P* Value
Binary variables							
Sex (female = 1)	19	(51.3)		248	(60.1)		.26
Cerebral abscess	37	(100)		0	(0)		
Clinical ischemic stroke	3	(8.1)		55	(13.4)		.41
Migraine	11	(29.7)		112	(27.5)		.51
Multiple PAVMs	28	(75.7)		213	(52.2)		.016
Definite HHT	34	(91.9)		368	(90.2)		.62
Use of oral iron	10	(27)		109	(26.7)		.63
Use of intravenous iron	4	(10.8)		14	(3.4)		.018
Use of blood transfusions	4	(10.8)		23	(5.6)		.16
Cerebral hemorrhage	0	(0)		3	(0.73)		.6
Liver transplantation for hepatic AVM	0	(0)		1	(0.25)		.76
Smoking history	8	(21.6)		92	(22.5)		.98
High blood pressure	5	(13.5)		39	(9.56)		.39
Venous thromboemboli	4	(10.8)		14	(3.43)		.022
Diabetes mellitus	1	(2.7)		9	(2.2)		.86
Continuous variables	No.	Median (Q1, Q3)	Range	No.	Median (Q1, Q3)	Range	
Age, y	37	50 (36, 62)	19–76	408	48 (34, 62)	16–89	.6
Oxygen saturation at abscess, %	34	92.1 (89, 95)	74–98	398	95.0 (91.8, 96.3)	72–99	.0016
Hemoglobin, g/L	35	144 (127, 158)	74–203	391	140 (125, 155)	59–201	.39
Arterial oxygen content, mL/dL	34	17.9 (13.8, 19.2)	9.4–21.3	385	17.5 (15.9, 19.2)	7.6–24.3	.75
Platelet count, ×10^9^/dL	34	252 (208, 295)	138–502	371	261 (221, 307)	70–606	.38
Platelet volume, fL	32	10.9 (10.3, 11.4)	9.5–13	367	10.6 (10, 11.2)	4.3–13.8	.04
Prothrombin time, sec	32	11.0 (10.6, 11.4)	9.9–22.8	367	10.7 (10.4, 11.2)	9–38.1	.037
Activated partial thromboplastin time, sec	32	26.3 (25.5, 28.3)	19.3–36.5	365	26.2 (24.7, 28.1)	13.2–43	.53
Fibrinogen, g/L	32	3.1 (2.47, 3.59)	1.47–4.65	361	3.03 (2.56, 3.58)	1.55–7.16	.8
C-reactive protein, IU/mL	30	2 (1.4, 4)	0.2,21	354	2.0 (1.2, 3.8)	0–118.2	.66
Serum iron, umol/L	33	15 (8, 22)	2.0–277.0	366	14 (8, 19)	0–64	.25
Transferrin saturation index, %	33	27 (13, 36)	0–100	365	21 (11, 29)	0–89	.18
Ferritin, μg/L	31	34 (20, 83)	3–151	342	34 (15, 73)	0–1795	.55
Largest PAVM feeding artery diameter, mm	25	5 (4, 6)	1–10	307	5 (2, 6)	0–14	.58
Pulmonary artery pressure, mean, mm Hg	25	13 (12, 16)	5–28	213	14 (12, 16)	6–50	.51

Range of interval to abscess: 9 months preabscess to 480 months postabscess (median, 9 months [interquartile range, 4–9 months] postabscess). Use of presentation data did not materially affect the respective ranks or *P* values (Supplementary Table 2).

Abbreviations: AVM, arteriovenous malformation; HHT, hereditary hemorrhagic telangiectasia; PAVM, pulmonary arteriovenous malformation.

**Table 5.** Multiple Logistic Regression Analyses of Cerebral Abscess Risk

Risk Factor	Odds Ratio	95% Confidence Interval	*P* Value
Oxygen saturation	0.895	.836–.958	.001
Male sex	2.625	1.18–5.86	.019
Transferrin saturation index	1.026	1.002–1.049	.034
Intravenous iron	5.423	1.397–21.06	.015
Venous thromboemboli	3.848	1.012–14.63	.048

This model of 380 individuals explained 12.2% of the variance of cerebral abscess (*P* = .0001). The model, and the preceding univariate analyses, were robust to the exclusion of the tiniest pulmonary arteriovenous malformations that may have been overreported on computed tomography (Supplementary Table 3), and to the inclusion or exclusion of venous thromboemboli (deep venous thromboses and/or pulmonary emboli; data not shown).

For SaO_2_, there was no relationship with higher-order variables, suggesting a relatively linear relationship between low SaO_2_ and abscess risk. Thus, for each 1% rise in SaO_2_, for example after embolization, the risk of brain abscess would reduce by 10.47% (95% CI, 4.18%–16.36%).

Males with PAVMs were at higher risk of cerebral abscess, estimated as 2.63-fold (95% CI 1.18- to 5.86-fold) higher, once adjusted for other variables in the final model. In this series, VTE was likely a result of the cerebral abscess (Table 2), but in other patients reviewed by us [8, 28], VTE could predate the abscess by several years. VTE could be partially replaced in the model by platelet volume, which in turn was associated with age and other hematological variables (data not shown).

**Figure 3.**
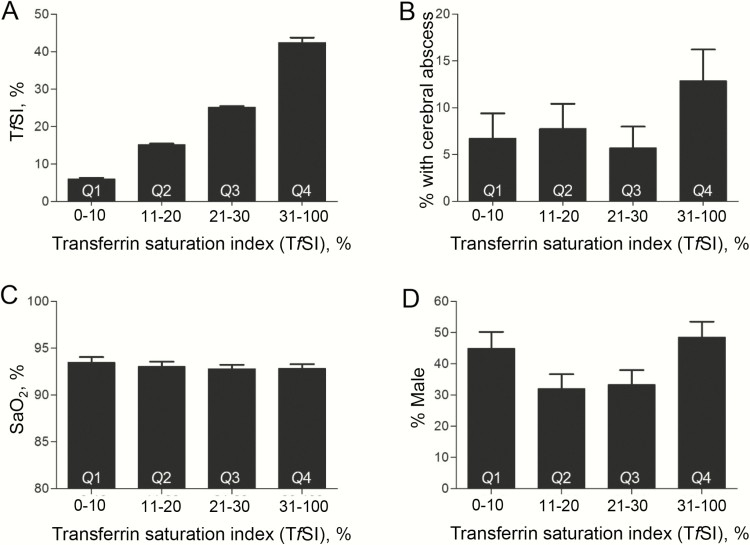
Variation of parameters across the transferrin saturation index (*Tf*SI) quartiles. *A*, *Tf*SI (%), where the normal institutional range was 20%–40%. Note that because boundary values were allocated to a single quartile, the exact numbers across *Q*1–*Q*4 were 89, 103, 105, and 101, respectively. *B*, Percentage of patients with cerebral abscess. *C*, Oxygen saturation (SaO_2_) where normal is ≥96%*. D*, Gender (% male). In all graphs, bars indicate mean and standard error of the mean. Across *Q*1–*Q*4, intravenous iron rates were 7.9%, 1.9%, 2.9%, and 5.0%, and venous thromboembolus rates were 3.4%, 7.8%, 5.7%, and 1.0%, respectively.

Unexpectedly, cerebral abscess risk was greater in patients who used intravenous iron (odds ratio, 5.82 [95% CI, 1.40–21.0]) and in patients with higher T*f*SI (with and without adjustment for intravenous iron administration). The T*f*SI odds ratio of 1.03 (95% CI, 1.00–1.05) related to each 1% change in a variable that ranged from 0–100% in the study cohort (Figure 3A). The enhanced risk appeared to lie within the upper T*f*SI quartile (Figure 3B), with no evident patterns in SaO_2_, sex, intravenous iron use, or deep venous thrombosis that might have enhanced the T*f*SI upper quartile risk (Figure 3C and 3D; Supplementary Table 4). Ninety-two patients had supranormal T*f*SI (>40%) and a cerebral abscess rate of 11.9%. Of this group, 62 (67.4%) were achieving a supranormal T*f*SI without ever having used supplementary iron or having received blood transfusions. Twenty-nine (31.5%) were using oral iron, with elemental iron contents ranging from 14 mg to 1000 mg (median, 65 mg) of elemental iron per day. Three (3.2%) were using intravenous iron, and 5 (5.5%) had been transfused.

T*f*SI concentrations define iron toxicity risks in the iron overload disorder hemochromatosis, with T*f*SI >50% considered a threshold for phlebotomy to remove body iron [29]. Eleven of 113 (9.7%) patients using iron reached this threshold, compared to 8 of 273 (2.9%) of patients not using iron or transfusions (*P* = .0084).

## DISCUSSION

We have shown, in a current UK population, that approximately 1 in 16 individuals with PAVMs experienced a cerebral abscess by median age 48 years, usually before their PAVMs were diagnosed. Substantial morbidity and healthcare burdens resulted. Bacterial isolates and interventional histories implicated endovascular dissemination of blood-borne organisms, often of odontogenic origin. Cerebral abscess risks were greater for patients with lower SaO_2_; for patients with T*f*SI in or above the high normal range; for males; and for those using intravenous iron.

Strengths of the study include the large numbers studied, comparing favorably with recent multicenter series evaluating cerebral abscesses in the general population [1, 7, 30]. Study methodology was validated by the replication of earlier identified associations between PAVM-induced cerebral abscess and specific microbial species, interventional histories, VTE, and male sex [8, 21, 31]. A valuable feature of the current cohort is the long-standing and systematic capture of SaO_2_ and iron-related variables that enabled identification of novel associations with likely pathogenic relevance. The main study weakness is that abscess histories were not captured prospectively, but such a study design is unrealistic for a condition such as PAVMs, which are normally diagnosed long after emergency neurosurgery and primary postoperative care. This explains the unusually high 100% survival rate [7] that reflected both the bias due to survival to the time of PAVM service review (n = 29) and, potentially, education and preventive measures following review. However, the study design meant that a higher proportion of the abscess group had blood tests after embolization, and that 7 of 16 interventional assessments were made retrospectively (in 2 cases, at an interval of >1 year), with the potential for recall bias. Additionally, whereas for previously treated patients, indices such as SaO_2_, hemoglobin, and iron datasets were only included where there was no known change in bleeding, PAVM, or iron use status between abscess and index capture, we cannot completely rule out uncaptured changes.

Within these limitations, the strength of the association with low SaO_2_, and absence of associations with PAVM number, or larger feeding artery size as previously proposed, is compelling. They support a more logical understanding that abscess risk relates to the proportion of blood flowing through the right-to-left shunts, and not anatomic attributes of individual PAVM structures. Higher T*f*SI and intravenous iron use in part reflected the significant iron deficiency anemia present in the PAVM/HHT cohort (Table 3): While the primary results from excessive blood losses in HHT are low serum iron, low T*f*SI, and low ferritin [20], following iron treatments, serum iron and T*f*SI rise rapidly (within 2 hours in healthy volunteers [32], sustained in HHT patients [33]), before a later increment in serum ferritin [32]. Thus iron deficiency treatments appeared to be inadvertently placing patients with prior iron deficiency into at least transient iron overload states.

The study raises intriguing questions regarding why higher circulating iron loads are associated with greater cerebral abscess risk. It is unlikely that patients with an unsuspected, enhanced risk of a cerebral abscess were more likely to be prescribed iron. An alternate possibility relates to enhanced survival of bacteria in blood and extracellular tissue fluids: Increased iron stores correlate with greater frequency and severity of many bacterial infections [34]. There have been suggestions that exogenous administration of transferrin may be an appropriate chelation therapy to reduce infection risks [34], but this seems counterintuitive as some microorganisms can liberate iron from transferrin [35, 36]. In view of the importance of blood–brain barrier injury in cerebral abscess pathogenesis [7, 37], potential endothelial injury by iron [32, 38] may also be relevant. More generally, why PAVMs should be such a risk factor for cerebral abscess is not intuitively clear. There is no obvious mechanical reason why endovascular bacteria should pose a greater threat if a 7–10 μm aperture filter is impaired. One possibility would be the passage of infected thromboemboli [37], but another relates to the potential for opsonized dormant bacteria to be reactivated, particularly if part of multicellular aggregates. Dental extractions and everyday activities such as toothbrushing can lead to endogenous dental bacteremias which, in the general population, are cleared (in terms of positive cultures) within minutes in the absence of antibiotics, and prevented or resolved earlier with prior antibiotic administration [23]. It is now known that similar bacterial species can be cultured from the bloodstream following later reactivation of dormant bacteria [39], and that iron can be one such activating agent [40].

The current findings should be helpful in several clinical settings: First, PAVM screening would appear to be an important part of the diagnostic paradigm for immunocompetent patients who have experienced a cerebral abscess. Second, the PAVM findings may be applicable to people with other forms of right-to-left shunts, particularly patients with congenital heart disease where hypoxemia ranges from profound (cyanotic congenital heart disease), to minimal, as for most cases of patent foramen ovale. The most direct applications are for known PAVM patients who are already advised to maintain scrupulous dental hygiene and use antibiotic prophylaxis prior to dental and surgical procedures. The identification of a higher-risk PAVM cohort with persistent hypoxemia and normal-high T*f*SI offers the opportunities for further targeted PAVM management. For such patients, including patients who previously had PAVM treatments, it would seem prudent to further optimize SaO_2_ improvements by embolization and potentially surgical methods, to avoid the 5.4-fold increased cerebral abscess risk currently implied for intravenous iron use, and to consider whether intravenous antibiotic prophylaxis [23], dental clearance, or other maneuvers should be employed to prevent dental bacteremias.

In conclusion, asymptomatic individuals with PAVMs remain at high risk of cerebral abscess and may account for significant proportions of immunocompetent patients presenting with odontogenic cerebral abscess. Study findings suggest caution in the use of intravenous iron, and potentially more extensive treatments for PAVM patients where iron loading is physiologically or pharmacologically inevitable.

## Supplementary Data

Supplementary materials are available at *Clinical Infectious Diseases* online. Consisting of data provided by the authors to benefit the reader, the posted materials are not copyedited and are the sole responsibility of the authors, so questions or comments should be addressed to the corresponding author.

## Supplementary Material

Supplementary_Data

## Notes

***Author contributions.*** E. J. B. reviewed the cerebral abscess patient case notes and performed preliminary analyses on the cerebral AVM cohort. S. B. and H. C. T. assisted with patient evaluations. K. B. B. contributed to microbiological discussions. J. E. J. reviewed patients, performed embolizations, and made all angiography associated measurements. C. L. S. designed the study, reviewed the patients, completed and validated the databases, performed all analyses, and wrote the manuscript. All authors contributed to manuscript review and approved the final version.

***Acknowledgments.*** The authors express their thanks to their colleagues for their support of the service, particularly in the pulmonary function laboratory, computed tomography scanner and angiography suites, and pathology laboratories at Hammersmith campus; and Drs John Livesey, Vatshalan Santhirapala, and Basel Chamali, who contributed to earlier database generation.

***Financial support.*** This work was supported by donations from hereditary hemorrhagic telangiectasia families, particularly the Thomas and Donald Ralph Memorial Fund. E. J. B. was supported by an Undergraduate Research Opportunity Bursary provided by Imperial College London. C. L. S. acknowledges the support of the National Institute for Health Research Imperial Biomedical Research Centre.

***Potential conflicts of interest.*** All authors: No reported conflicts of interest. All authors have submitted the ICMJE Form for Disclosure of Potential Conflicts of Interest. Conflicts that the editors consider relevant to the content of the manuscript have been disclosed.
