# Supplementary material for: Cerebral Abscess Associated With Odontogenic Bacteremias, Hypoxemia, and Iron Loading in Immunocompetent Patients With Right-to-Left Shunting Through Pulmonary Arteriovenous Malformations
Source: Clin Infect Dis. 2017 Apr 19;65(4):595–603. doi: 10.1093/cid/cix373 (PMC5849101; doi:10.1093/cid/cix373)
Supplement: Supplementary_Data [file cix373_suppl_supplementary_data.docx]

**Cerebral abscess associated with odontogenic bacteremias, hypoxemia, and iron loading in immunocompetent patients with right-to-left shunting through pulmonary arteriovenous malformations**

**Boother EJ, Brownlow S, Tighe HC, Bamford K, Jackson JE, Shovlin CL**

**DATA SUPPLEMENT**

## **SUPPLEMENTARY METHODS**

***Study Cohort***

The cohort represents 445 consecutive adult PAVM patients with CT-scan confirmed PAVMs (including 403 (90.5%) with hereditary haemorrhagic telangiectasia) reviewed between June 2005 and December 2016 at a single institution in the UK [1]. As detailed below, based on existing protocols and a systematic literature review [2], from 1999, individuals presenting with PAVMs and/or definite or possible HHT [3] underwent detailed same-day clinical assessments. The current cohort of patients with PAVMs were therefore identified through diagnoses made prior to referral; through investigation of patients with symptoms suggestive of PAVMs; or through the PAVM screening service offered to patients with definite or possible HHT [3].

***Clinical assessments for patients with known or suspected PAVMs***

General clinical variables defined at the time of initial clinic assessment included a full HHT symptomatic review including nosebleed severity, other blood losses, and dietary iron intake.[4] The documented presence of telangiectasia or AVMs on earlier endoscopic or imaging analyses was noted. A full drug history was recorded with a particular focus on treatments used for HHT haemorrhage (in this series, female hormones, tranexamic acid, thalidomide, bevazicumab and iron/blood replacement (oral and intravenous iron; blood transfusions)). Patients were asked to bring their medications to clinic, and the drug history was recorded with reference to actual versus prescribed usage. Iron treatment side effects [5], and stated allergies to antibiotics and other drugs were also recorded in all cases.

In all cases, a full past medical history including hypertension on treatment; cardiac arrhythmias; and known diabetes mellitus, hypercholesterolemia or cardiac disease; pregnancy histories; family histories; and current or ex-smoking status. For PAVM complications, strokes were defined as clinically evident, focal cerebral deficits of rapid onset, at least 24hs in duration,with a report of a causative infarct on clinical post stroke cross-sectional imaging required to assign ischemic aetiology[6]. Cerebral abscesses were diagnosed by the requirement for neurosurgical drainage. Headaches were defined as migrainous for patients on migraine treatment, or describing recurrent headaches with aura and/or teichopsia.[7]

Clinical examination included evaluation of HHT telangiectasia on lips, tongue, oral mucosa, palate, finger tip pads and ears; signs of right-to-left shunting (cyanosis, clubbing), general cardiorespiratory, abdominal and neurological examinations, and a simple inspection of dental hygiene.

Pulmonary function testing measured spirometric values (forced expiratory volume in one second (FEV1), vital capacity (VC), and diffusion capacity for carbon monoxide uncorrected (TLCO), and per unit of alveolar volume (KCO). CT scans were performed, if not performed previously, to identify PAVMs, and define anatomic suitability for embolisation. Oxygen saturation (SaO_2_) was measured by pulse oximetry in the erect posture for 10 minutes, with the mean value of minutes 7-10 recorded for serial evaluations, since this better reflects right-to-left shunt size than SaO_2_ in other postures,[8,9] and is more relevant for patients’ daily activities.

All PAVM patients, and all patients with known or suspected HHT, were also offered screening/investigation for iron deficiency anemia (using complete blood counts, serum iron, transferrin saturation index (T*f*SI) and ferritin); coagulopathies using prothrombin time (PT), activated partial thromboplastin time (APTT) and fibrinogen; and routine biochemical indices including renal function, liver function tests and C-reactive protein. Complete (full) blood counts were measured on XE Series Analysers (Sysmex, UK) and biochemical indices on Ci1600 Architect Analysers (Abbott Diagnostics, Ireland). Patients with symptoms suggestive of other HHT-related visceral AVMs underwent formal investigations, but screening of asymptomatic HHT/PAVM patients for other AVMs was restricted to selected cases.

At our institution, all patients with PAVMs received written advice on optimising dental hygiene and advice to use prophylactic antibiotics prior to dental and surgical procedures [10], in addition to general and if relevant, HHT medical advice. Where indicated, PAVM embolization was performed as described.[11,12] The usual reason why no treatment was offered was the technical non feasibility of treating multiple PAVMs with feeding arteries of 2mm diameter or less. The diameter of the feeding artery to the PAVM sac was measured by thoracic CT scan, and/or at the time of angiography. Other reasons for non-intervention included patient choice, and presence of severe pulmonary arterial hypertension identified by prior echocardiography. For patients undergoing embolization, pulmonary artery pressures (PAP) were recorded by a centrally-placed catheter prior to contrast injection.[11,12]

Follow up was recommended at our institution for all patients who had been treated by embolization, and comprised a clinical, radiographic (usually chest x-ray), physiological (particularly SaO_2_/pulse) and blood based assessment approximately 6 months after embolization, and at subsequent time points as required. General results are presented elsewhere for earlier patients in the series.[6,13,14] Variable follow up advice was provided for patients first treated at other institutions.

For the cerebral abscess cohort, in some cases, follow-up assessment indices were the most relevant to the cerebral abscess, and were utilised for the purposes of the current study. With one exception (a patient who had previously declined first treatment), patients experiencing a cerebral abscess underwent a further CT scan. In all except one case, an attempt to perform further embolization was recommended as detailed in the text, even if the diameter of the feeding arteries was at or below the limits of what would usually be treated. As a result, ‘PAVM embolization’ was not considered a robust variable to use in logistic regression, since the decision to attempt embolization of smaller (feeding artery diameter <<3mm) PAVMs was substantially influenced by the prior occurrence of a cerebral abscess.

**PAVM screening for patients with known or suspected HHT**

All patients with known or suspected HHT who had no prior PAVM screen in adult life, underwent general assessment at our institution as described above. The PAVM screening protocol employed contrast echocardiography if SaO_2_ were normal, there was a low suspicion of risk for PAVMs or HHT, or following patient choice. Otherwise, in view of the very high positive rates for contrast echocardiography in HHT (87% for *ENG* [15], the most common HHT genotype in our population), a single thoracic CT scan was performed if not performed to identify PAVMs, define anatomic suitability for embolisation, and for unilateral disease, to allow subsequent selective angiography to be limited to a single side and limit radiation exposure.

The overall population were from across the UK, where there have been recent epidemiological studies demonstrating general rates of HHT diagnoses and complications.[16,17] At our clinical service, in keeping with the Curaçao criteria,[3] a diagnosis of HHT was made in the presence at least three of epistaxis, characteristic telangiectasia, visceral involvement (such as pulmonary, cerebral or hepatic AVMs, or gastrointestinal telangiectasia) or affected first degree family member, or a positive HHT gene test. None of the wider HHT cohort without PAVMs experienced a cerebral abscess.

***Details of Earlier Diagnoses and Treatments***

In the full cohort, 31 had received treatments at other institutions. 6 patients (4 [10.8%] abscess, 2 [0.98%] non abscess) had received surgical resections elsewhere at a range of 7-40 (median 20) years prior to referral. 25 patients ((7 [18.9%] abscess and 18 [4.4%] non-abscess) had received embolisations elsewhere at a range of 2-204 (median 4.5) months prior to referral. Unless pre-treatment data were recorded for these individuals, SaO_2_, hemoglobin and red cell indices were recorded as unknown, to minimise bias by assigning spuriously normal indices to a patient who may have previously had decades of life with untreated PAVMs and different risk factor status, to the time of presentation and assessment at our institution.

In addition to diagnoses made as a result of investigations post abscess, reasons why cerebral abscess patients had received their diagnosis of PAVMs included investigations of incidental hypoxemia (N=3), HHT screens (N=2), dyspnea (N=1), hemoptysis (N=1), transient ischemic attacks (N=1), and polycythemia (N=1).

## ***Data Analyses***

Statistical analyses were performed using STATA IC version 13 (Statacorp, Texas) to generate descriptive statistics, to compare the two data sets using Mann Whitney rank test, and to perform regression analyses. GraphPad Prism 5 (Graph Pad Software Inc, San Diego) was used for additional analyses in the cerebral abscess cohort, and to generate T*f*SI quartile graphs.

In logistic regression using cerebral abscess as the outcome (dependent) variable, 34 variables were tested stepwise, first in univariate analyses (HHT, age, gender, SaO_2_, CaO_2_, single/multiple, largest PAVM feeding artery diameter, red cell indices relevant to iron status (hemoglobin, hematocrit, red cell number, mean corpuscular volume (MCV), mean corpuscular haemoglobin (MCH), mean corpuscular haemoglobin concentration (MCHC)); indices of active bleeding (red cell distribution width (RDW) and platelets; platelet volume; acute phase response indices C-reactive protein and fibrinogen; coagulation indices PT and APPT; mean pulmonary artery pressure (PAP); serum iron; T*f*SI, ferritin; use of oral iron; use of intravenous iron; use of blood transfusions; smoking; high blood pressure; venous thromboemboli (VTE, i.e. deep venous thrombosis or pulmonary emboli); diabetes mellitus; migraines and ischemic stroke). To minimise the possibility of bias due to inclusion of smaller CT lesions that were in fact not PAVMs, univariate analyses were also performed restricting to “embolized only”, and “absolute certainty” subgroups: As relationships were materially unchanged by restricting the population in either way (other than reducing the N numbers and magnitude of *p*-values), the full population was used for further analyses.

SaO_2_ demonstrated the strongest univariate association. SaO_2_-adjusted odds ratios were calculated by adding each variable to separate models which were simultaneously examining the associations of cerebral abscess with SaO_2_. With the emergence of higher T*f*SI in SaO_2_-adjusted regression analyses, and appreciation of the risk distribution profile (Figure 2), case notes were cross checked to capture the exact oral and intravenous iron intakes for patients in the upper quartile at the time of the measured index. Iron indices demonstrated the strongest relationships in SaO_2_-adjusted models, and were examined simultaneously, demonstrating that T*f*SI and intravenous iron were both required to maximise the proportion of overall variance described by the model. Further variables were tested to generate the final model that explained the greatest proportion of variance (Table 5). The model explaining the greatest proportion of biological variability was cross checked against “absolute certainty” subgroups and higher order variables.

# **SUPPLEMENTARY DATA**

**Supplementary Table 1: Cohort Characteristics of the 37 cerebral abscess patients**

| **Continuous variables** | **N** | **Median (Q1,Q3)** | **Range** |  | **Binary variables** | **Total** | **Number** | **%** |
| --- | --- | --- | --- | --- | --- | --- | --- | --- |
| Age at abscess (yr) | 37 | 50 (36,62) | 19-76 |  | Pulmonary arteriovenous malformations | 37 | 37 | 100 |
| Largest PAVM f.a.d (mm) | 25 | 5 (4,6) | <2-10 |  | Multiple PAVMs (%) | 37 | 29 | 78.3 |
| White cell count (x10^9^/dl) | 31 | 6.5 (5.3, 8.1) | 3.1-11 |  | Some PAVMs with f.a.d ≤ 3mm | 37 | 31 | 83.7 |
| Neutrophils (x10^9^/dl) | 31 | 3.8 (2.8, 4.8) | 1.9-8.2 |  | All PAVMs with f.a.d ≤ 3mm | 37 | 5 ^a^ | 13.5 |
| Lymphocytes (x10^9^/dl) | 31 | 1.8 (1.3, 2.6) | 0.4-4.7 |  | Embolisation received | 37 | 35 | 94.5 |
| Monocytes(x10^9^/dl) | 31 | 0.6 (0.4, 0.7) | 0.3-1.2 |  | Respiratory symptoms | 37 | 9 | 24.3 |
| Eosinophils (x10^9^/dl) | 31 | 0.1 (0.1, 0.2) | 0-0.6 |  | Reason for PAVM presentation | 37 | 3 | 8.1 |
| Basophils (x10^9^/dl) | 31 | 0 (0,0) | 0-0.1 |  | Dyspnea | 37 | 6 | 16.2 |
| C-reactive protein (iu/ml) | 384 | 2 (1.2, 3.85) | 0-118 |  | Hemoptysis | 37 | 1 | 2.7 |
| Fibrinogen (g/L) | 393 | 3.03 (2.56, 3.58) | 1.47-7.16 |  | Used supplementary home oxygen^b^ | 37 | 1 | 2.7 |
| Hemoglobin (g/L) | 35 | 144 (127, 158) | 74-203 |  | Venesected | 37 | 1 | 2.7 |
| Hematocrit (%) | 34 | 0.44 (0.41, 0.47) | 0.28-0.58 |  | Clinical ischemic stroke | 37 | 3 | 8.1 |
| Platelets (x10^9^/dL) | 34 | 252 (208, 295) | 138-502 |  | Migraines | 37 | 11 | 29.7 |
| Oxygen saturation, SaO_2_ (%) | 34 | 92 (89,95) | 74-98 |  | Definite HHT | 37 | 34 | 91.9 |
| Arterial oxygen content (CaO_2_, mls/dL) | 34 | 17.8 (13.8, 19.3) | 9.4-21.3 |  | Use of oral iron | 37 | 10 | 27 |
| CaO_2_ (mls/dL) if SaO_2_ ≥ 95% | 13 | 16.2 (13.0, 18.3) | 9.4-20.1 |  | Use of intravenous iron ^c^ | 37 | 4 | 10.8 |
| CaO_2_ (mls/dL) if SaO_2_ <95% | 21 | 17.9 (17.0, 19.6) | 12.6-21.3 |  | Use of blood transfusions | 37 | 4 | 10.8 |
| CaO_2_ (mls/dL) if SaO_2_ <90% | 10 | 17.3 (13.8, 19.8) | 12.6-20.7 |  | Cerebral hemorrhage | 37 | 0 | 0 |
| Prothrombin time (s) ^d^ | 32 | 11.0 (10.6, 11.4) | 9.9-22.8 |  | Liver transplantation for hepatic AVMs | 37 | 0 | 0 |
| Activated partial thromboplastin time (s)^e^ | 32 | 26.3 (25.5, 28.25) | 19.3-36.5 |  | Current or former smoker | 37 | 8 | 21.6 |
| Serum iron (umol/l) | 33 | 15 (8, 22) | 2-277 |  | Hypertension | 37 | 5 | 13.5 |
| Serum iron, (umol/l) if using iv iron ^f^ | 4 | 25 (13, 153.5) | 6-277 |  | Venous thromboemboli (VTE) | 37 | 4 | 27 |
| Serum iron, (umol/l) if not using iv iron | 27 | 15 (8, 22) | 2-40 |  | VTE in 6 months post cerebral abscess | 37 | 4 | 27 |
| Transferrin saturation index (T*f*SI, %) | 33 | 27 (13, 36) | 0-100 |  | VTE in other period | 37 | 0 | 0 |
| TfSI, (%) if using iv iron‡ | 4 | 39.5 (21, 72.5) | 8-100 |  | Diabetes mellitus | 37 | 1 | 2.7 |
| TfSI, (%) if not using iv iron | 27 | 27 (13,36) | 2-57 |  |  |  |  |  |
| Ferritin (μg/l) | 29 | 34 (22,82) | 3-151 |  |  |  |  |  |
| Pulmonary artery pressure (mean), mmHg | 25 | 13 (12, 16) | 5-28 |  |  |  |  |  |

**Legend:** f.a.d. feeding artery diameter. ^a^ One patient had a significant patent foramen ovale as a potential alternative conduit for paradoxical emboli. ^b^Discontinued as no benefit noted. ^c^ and ^f^ : 3 cases were also using blood transfusions and the serum iron and T*f*SI distributions were similar for the two groups. ^d^ and ^e^  One individual using warfarin

# **Supplementary Table 2: Presentation data comparisons**

|  | **Number** | **Median (Q1,Q3)** | **Range** | **Number** | **Median (Q1,Q3)** | **Range** |  |
| --- | --- | --- | --- | --- | --- | --- | --- |
| CLOSEST TO ABSCESS (AS IN TABLE 4) |  |  |  |  |  |  |  |
| Oxygen saturation SaO_2_ (%) | 34 | 92.1 (89, 95) | 74-98 | 398 | 95.0 (91.8, 96.3) | 72-99 | **0.0016** |
| Hemoglobin (g/L) | 35 | 144 (127, 158) | 74-203 | 391 | 140 (125, 155) | 59-201 | 0.39 |

|  | **Number** | **Median (Q1,Q3)** | **Range** | **Number** | **Median (Q1,Q3)** | **Range** |  |
| --- | --- | --- | --- | --- | --- | --- | --- |
| PRESENTATION |  |  |  |  |  |  |  |
| Oxygen saturation SaO_2_ (%) | 32 | 91.25 (87, 95) | 74-98 | 386 | 95.0 (91.3, 96.3) | 72-99 | **0.0016** |
| Hemoglobin (g/L) | 21 | 144 (122, 158) | 74-203 | 391 | 140 (124, 154) | 59-287 | 0.57 |

Legend: Key indices as evaluated A) At the time-point closest to the abscess (as presented in Table 4), and B) At initial presentation. Note that in these rank-based measurements, there was no material change in SaO_2_ rankings (between abscess cases versus no abscess) and hence the p-value was unchanged.

# **Supplementary Table 3:** **Multiple logistic regression analyses of cerebral abscess risk in restricted population.**

#

|  | | **Odds ratio** | | **95% confidence intervals** | | **P value** | |
| --- | --- | --- | --- | --- | --- | --- | --- |
| SaO_2_ (%) | | 0.895 | | 0.836, 0.958 | | 0.001 | |
| Male gender | | 2.625 | | 1.18, 5.86 | | 0.019 | |
| Transferrin saturation index (T*f*SI) | | 1.026 | | 1.002, 1.049 | | 0.034 | |
| Intravenous iron use | | 5.423 | | 1.397, 21.06 | | 0.015 | |
| Venous thromboemboli (VTE) | | 3.848 | | 1.012, 14.63 | | 0.048 | |
|  | |  | |  | |  | |

**Legend:** This model of 380 individuals excluded the tiniest PAVMs that may have been over-reported on CT, and explained 12.2% of the variance of cerebral abscess (p= 0.0001).

# **Supplementary Table 4: Relationships between cerebral abscess and iron/ red cell indices by logistic regression**

|  |  | **SaO_2_ and gender adjusted^a^** | | | |  | **SaO_2_, gender, VTE and iv iron -adjusted** | | | | |  |
| --- | --- | --- | --- | --- | --- | --- | --- | --- | --- | --- | --- | --- |
|  | **N** | **OR** | **95% CI** | **p-value** | **model r^2^** |  | **N** | **OR** | **95% CI** | **p-value** | **model r^2^** | |
| Serum iron (ug/ml) | 391 | 1.02 | 1.00, 1.04 | 0.052 | 0.066 |  | 381 | 1.023 | 0.99, 1.060 | 0.212 | 0.12 | |
| T*f*SI (%) | 390 | 1.02 | 1.00, 1.04 | 0.058 | 0.054 |  | 380 | 1.025 | 1.00, 1.050 | 0.034 | 0.12 | |
| Ferritin (μg/l) | 365 | 1.00 | 0.99, 1.00 | 0.31 | 0.044 |  | 356 | 1 | 0.99, 1.00 | 0.15 | 0.12 | |
|  |  |  |  |  |  |  |  |  |  |  |  | |
| Hemoglobin(g/L) | 419 | 1.00 | 0.98, 1.01 | 0.58 | 0.035 |  | 407 | 1.01 | 0.99, 1.03 | 0.33 | 0.09 | |
| CaO_2_ (mls/dL) | 419 | 0.95 | 0.84, 1.07 | 0.37 | 0.037 |  | 407 | 1.05 | 0.91, 1.21 | 0.50 | 0.09 | |
| Hematocrit (%) | 400 | 0.77 | 0.00, 378 | 0.77 | 0.038 |  | 389 | 138.8 | 0.055, 3.5x10^5^ | 0.22 | 0.10 | |
| Red cell number (x10^9^/dL) | 400 | 1.08 | 0.57, 2.04 | 0.82 | 0.037 |  | 389 | 1.78 | 0.89, 3.54 | 0.101 | 0.11 | |
| MCHC (g/L) | 398 | 1.00 | 0.99, 1.01 | 0.48 | 0.040 |  | 387 | 1.00 | 0.99, 1.01 | 0.95 | 0.10 | |
| RDW (%) | 398 | 1.10 | 0.99, 1.23 | 0.075 | 0.049 |  | 387 | 1.03 | 0.90, 1.17 | 0.66 | 0.10 | |

**Legend:** OR, odds ratio; CI, confidence interval **a.** Iron, T*f*SI and ferritin unchanged by exclusion of gender from the model. CaO_2_, arterial oxygen content (mls/dL); MCHC, mean corpuscular hemoglobin concentration; RDW, red cell distribution width.

# **SUPPLEMENTARY REFERENCES**

1 HHT and PAVMs: <https://www.imperial.nhs.uk/our-services/respiratory-medicine/hht-pavms>. Accessed 14.03.2017

2. Shovlin CL, Letarte M. Hereditary haemorrhagic telangiectasia and pulmonary arteriovenous malformations: issues in clinical management and review of pathogenic mechanisms.Thorax. 1999 Aug;54(8):714-29.

3. Shovlin CL, Guttmacher AE, Buscarini E, Faughnan ME, Hyland RH, Westermann CJ, Kjeldsen AD, Plauchu H. Diagnostic criteria for hereditary hemorrhagic telangiectasia (Rendu-Osler-Weber syndrome). Am J Med Genet. 2000 Mar 6;91(1):66-7.

4. Finnamore HE, Whelan K, Hickson M, Shovlin CL. Top dietary iron sources in the UK. Br J Gen Pract. 2014 Apr;64(621):172-3.

5. Finnamore H, Le Couteur J, Hickson M, Busbridge M, Whelan K, Shovlin CL. Hemorrhage-adjusted iron requirements, hematinics and hepcidin define hereditary hemorrhagic telangiectasia as a model of hemorrhagic iron deficiency. PLoS One. 2013 Oct 16;8(10):e76516

6. Shovlin CL, Chamali B, Santhirapala V, et al. Ischaemic strokes in patients with pulmonary arteriovenous malformations and hereditary hemorrhagic telangiectasia: associations with iron deficiency and platelets. PLoS ONE, **2014** Feb 19;9(2):e88812

7. Elphick A, Shovlin CL. Relationships between epistaxis, migraines, and triggers in hereditary hemorrhagic telangiectasia. Laryngoscope. **2014** Jul;124(7):1521-8.

8 Ueki T, Hughes JMB, Peters AM, Bellingan GJ, Mohammed MAM et al. Oxygen and 99mTc-MAA shunt estimations in patients with pulmonary arteriovenous malformations: effects of changes in posture and lung volume. Thorax, **1994**; 49: 327-331;

9 Thompson RD, Jackson J, Peters AM, Doré CJ, Hughes JM. Sensitivity and specificity of radioisotope right-left shunt measurements and pulse oximetry for the early detection of pulmonary arteriovenous malformations. Chest, **1999**; 115: 109-13.

10 Shovlin CL, Bamford K, Wray D. Post-NICE 2008: Antibiotic prophylaxis prior to dental procedures for patients with pulmonary arteriovenous malformations (PAVMs) and hereditary haemorrhagic telangiectasia. Br Dent J. 2008 Nov 22;205(10):531-3. doi: 10.1038/sj.bdj.2008.978.

11 Gill SS, Roddie ME, Shovlin CL, Jackson JE. Pulmonary arteriovenous malformations and their mimics. Clin Radiol, **2015** ; 70(1): 96-110

12 Hart JL, Aldin Z, Braude P, Shovlin CL, Jackson JE. Embolization of pulmonary arteriovenous malformations using the Amplatzer vascular plug: successful treatment of 69 consecutive patients. Eur J Radiology, 2010; 20: 2663–70

13 Santhirapala V, Williams LC, Tighe HC, et al. Arterial oxygen content is precisely maintained by graded erythrocytotic responses in settings of high/normal serum iron levels, and predicts exercise capacity. An observational study of hypoxaemic patients with pulmonary arteriovenous malformations. PLoS ONE, **2014;** 9(3): e90777

14 Rizvi A, Macedo P, Babawale L, et al. Hemoglobin is a vital determinant of arterial oxygen content in hypoxemic patients with PAVMs. Ann Am Thorac Soc, **2017** Mar 7. doi: 10.1513/AnnalsATS.201611-872OC. [Epub ahead of print]

**15.** van Gent MW, Post MC, Snijder RJ, Westermann CJ, Plokker HW, Mager JJ. Real prevalence of pulmonary right-to-left shunt according to genotype in patients with hereditary hemorrhagic telangiectasia: a transthoracic contrast echocardiography study. Chest 2010; 138:833-9

16 Donaldson JW, McKeever TM, Hall IP, Hubbard RB, Fogarty AW. The UK prevalence of hereditary haemorrhagic telangiectasia and its association with sex, socioeconomic status and region of residence: a population-based study. Thorax, **2014**; 69(2): 161-7

17 Donaldson JW, McKeever TM, Hall IP, Hubbard RB, Fogarty AW. Complications and mortality in hereditary hemorrhagic telangiectasia: A population-based study. Neurology. **2015** May 5;84(18):1886-93
